# Phacoemulsification with Intraocular Implantation of Lens, Endocyclophotocoagulation, and Endoscopic-Goniosynechialysis (PIECES): A Combined Technique for the Management of Extensive Synechial Primary Angle Closure Glaucoma

**DOI:** 10.5005/jp-journals-10028-1243

**Published:** 2018-03-01

**Authors:** Pouya Alaghband, Ian AS Rodrigues, Saurabh Goyal

**Affiliations:** 1 Registrar, Department of Ophthalmology, St Thomas Hospital, London United Kingdom; 2 Consultant, Department of Ophthalmology, St Thomas Hospital, London United Kingdom; 3 Consultant, Department of Ophthalmology, St Thomas Hospital, London United Kingdom

**Keywords:** Angle closure, Cyclophotocoagulation, Glaucoma, Goniosynechialysis, Intraocular pressure.

## Abstract

Primary angle closure glaucoma (PACG) is more blinding (1 in 4 cases) than primary open angle glaucoma (1 in 10 cases). Cataract surgery is an effective initial treatment for majority of cases of PACG. However, cataract surgery alone may not be enough to control intraocular pressure (IOP) in cases with extensive synechial angle closure glaucoma. It is reported that glaucoma drainage surgery is needed in 12% of PACG cases after cataract surgery. Some experts combine cataract surgery with either goniosynechialysis (GSL) or endocyclophotocoagulation (ECP) to enhance IOP control. However, neither combination ensures complete success. We report three subjects with extensive synechia! angle closure in whom we facilitated a technique that combines lens extraction with ECP and endoscopic—GSL (PIECES). We demonstrated that this combined technique was a more effective and efficient method of achieving lower IOP in the presence of extensive synechial PACG. We believe that it addresses both the inflow and outflow of the aqueous humor simultaneously. Two out of three patients had good IOP control without medication and one patient needed one drop after a minimum 12 months of follow up. Furthermore, it may reduce the need for medical therapy and future more invasive glaucoma drainage surgery.

**How to cite this article: **Alaghband P, Rodrigues IAS, Goyal S. Phacoemulsification with Intraocular Implantation of Lens, Endocyclophotocoagulation, and Endoscopic-Goniosynechialysis (PIECES): A Combined Technique for the Management of Extensive Synechial Primary Angle Closure Glaucoma. J Curr Glaucoma Pract 2018;12(1):45-49.

## INTRODUCTION

It is estimated that approximately 20 million people will be affected by PACG by 2020 and just over 5 million individuals will be bilaterally blind from this.^1^ The recently published EAGLE study^2^ (effectiveness of early lens extraction for the treatment of PACG) has presented a paradigm shift in the management of PACG. It showed a clear benefit of primary lens extraction in controlling IOP and reducing the need for future glaucoma drainage surgery in PACG with any IOP and PAC with IOP > 30 mm Hg.

Another aspect of PACG, which needs to be addressed, is the presence of peripheral anterior synechiae (PAS) leading to synechial angle closure and trabecular mesh-work dysfunction. This may affect the long-term success of cataract surgery in controlling IOP. Combined phaco-emulsification and GSL^3,4^ has been shown to successfully control IOP in these types of PACG cases. Although the number of medications required to control IOP after phaco-GSL is shown to be significantly less than phaco alone,^4,5^ often it cannot eliminate the need for further IOP lowering medications or glaucoma drainage surgery.^6^

Synechialysis is conventionally performed using a direct goniolens but can also be carried out endoscopi-cally.^1,7^ The merits of the latter are the ability to directly visualize the angle structures under greater magnification and therefore improve the accuracy of the procedure.^8^ It also allows larger parts of the angle to be accessible for synechialysis using a bimanual technique. In addition, ECP has provided further options in glaucoma management as one of the few surgical treatments that reduces aqueous production.^8^ While ECP combined with cataract extraction is proven to be safe and effective in controlling IOP in glaucoma cases,^9^ patients may still rely on medical therapy to achieve their target IOP postoperatively.^10^ Persistent PAS are likely to increase the long-term dependence on glaucoma medication.

We report a technique called PIECES: Combining phacoemulsification with intraocular lens (IOL) implantation (PI), endocyclophotocoagulation, and endoscopic GSL to control IOP in extensive (>270°) synechial angle closure glaucoma. This approach tackles both the inflow and outflow of the aqueous humor simultaneously. Consequently, it has the potential to minimize the need for glaucoma medications and further glaucoma drainage surgery in a single operation. This procedure also preserves the conjunctiva so that future drainage surgery can still be performed if required.

The Ethics Committee deemed ethical approval unnecessary for this study, considering that we performed each procedure separately routinely in our department. We have treated three eyes of three patients with this technique. All patients had ≥270° of PAS, patent peripheral iridotomies, and uncontrolled IOP of >25 mm Hg despite at least three glaucoma medications, including oral acet-azolamide in two patients. One subject had recent acute primary angle closure (APAC) and the other cases were diagnosed with PACG with moderate glaucoma.

All patients were seen 1 day, 1 week, 1 month, and then every 3 to 6 months after surgery. At each visit they had visual acuity, slit lamp biomicroscopic examination, gonioscopy, Goldmann applanation tonometry, and oph-thalmoscopy performed.

## SURGICAL TECHNIQUE

All surgeries were performed by a single experienced glaucoma surgeon (SG). After sub-Tenon’s local anesthesia, a clear corneal incision (2.4 mm) was followed by a side port positioned at 90° to the main incision and the injection of viscoelastic (Healon®, OVD, Abbott Medical Optics Inc., USA). Capsulorrhexis and hydro-dissection was then performed. The lens was removed by phacoemulsification of the lens nucleus and aspiration of the cortical lens matter. After further injection of the viscoelastic (Healon^®^, OVD, Abbott Medical Optics Inc., USA), an acrylic injectable IOL (AcrySof® SA60AT, Alcon, Texas, USA) of the appropriate power was inserted into the capsular bag. Once the IOL was positioned in the bag and the pupil was still dilated, viscoelastic (Healon^®^, OVD, Abbott Medical Optics Inc., USA) was injected in the ciliary sulcus between the iris and the IOL to allow access to the ciliary processes. The 20-G ECP probe (E4; Endo Optiks, Little Silver, New Jersey, USA), containing an endoscope, an illumination source, the diode LASER (810 nm), and a helium-neon aiming beam, was then inserted into the anterior chamber via the clear corneal incision. Photocoagulation of the ciliary processes was performed with direct visualization on a monitor connected to the endoscope. Treatment energy was 0.25 to 0.4 W and the treatment duration was set to continuous. Treatment was deemed sufficient if a characteristic whitening and contraction of the ciliary process was observed. Care was taken to avoid “popping” of ciliary processes. If the ciliary process is observed to “pop,” this implies treatment energy is too high and/or the treatment distance (distance from endoscope tip to ciliary tissue) is too short; appropriate adjustment is then made by withdrawing the probe back or reducing the power. Treatment was applied to ciliary processes throughout 360° via two to three clear corneal incisions (by making an extra incision if required opposite to the side port).

The pupil was then constricted by intracameral injection of carbachol (Miochol-E; Bausch & Lomb, New Jersey, USA) in the anterior chamber and the angle was hyperin-flated with Healon sequentially to enhance the visualization and to reduce the risk of inadvertently touching the corneal endothelium while performing GSL. The same endoscope was then inserted into the anterior chamber and advanced forward to the anterior chamber angle. Preexisting ports were utilized to sequentially gain access to the majority of the trabecular meshwork. The PAS in the angle were gently stripped down using a blunt cyclodi-alysis spatula or iris repositor under direct visualization.

**Figs 1A and B:**
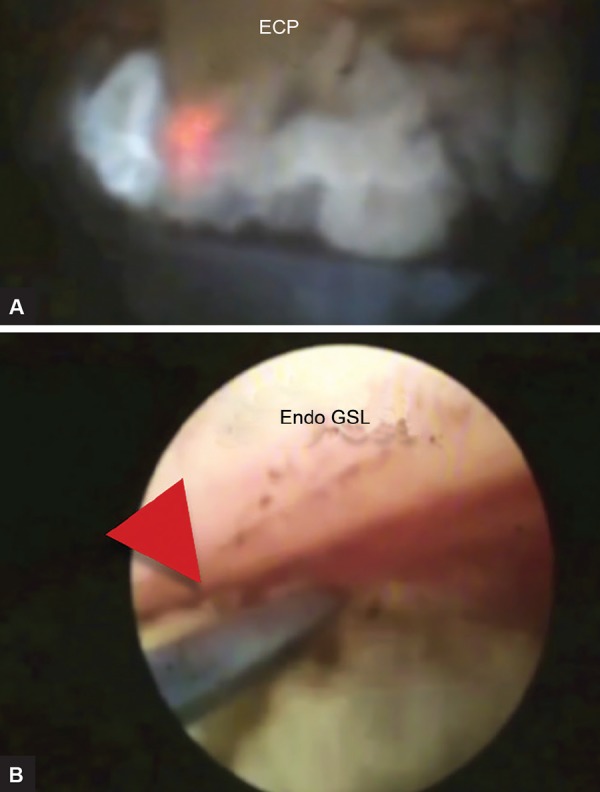
Intraoperative view of ECP and endo-GSL, the red arrow head is pointing to PAS in the angle

**Figs 2A and B:**
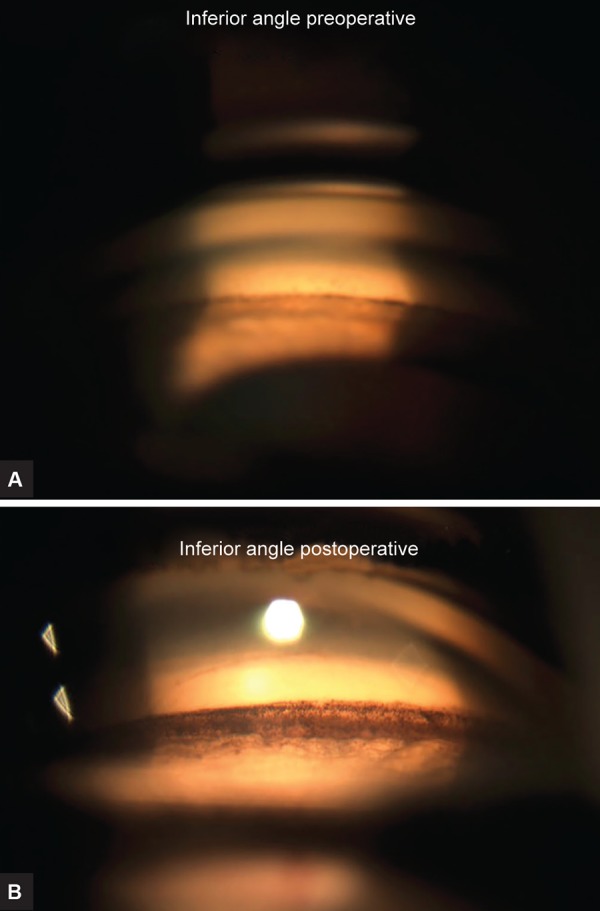
Gonioscopic view pre- and postoperatively

The viscoelastic was then thoroughly washed out. The larger corneal incisions were sutured using 10.0 nylon suture that were removed after 1 to 2 weeks. Intracameral cefuroxime (0.1 mg/0.1 mL) and dexamethasone (0.2 mL) were given at the end of surgery. Immediately following surgery, glaucoma medications to the treated eye were stopped. Patients were given g. chloramphenicol QID for 2 weeks and g. dexamethasone preservative-free q2h for 1 week and then tapered over the next 4 to 6 weeks. Pre-, intra-, and postoperative images are illustrated in Figures 1 and 2.

## RESULTS

We have treated three eyes of three patients with this technique. All patients had ≥270° of PAS and uncontrolled IOP >25 mm Hg, despite at least three glaucoma medications, including oral acetazolamide in two patients. Following PIECES, IOP was successfully controlled (success was defined as IOP reduction (≥30% IOP reduction) (Table 1) in all cases with 1 eye drop required after a month postoperatively in only 1 patient. We encountered no intra—and postoperative complications during the follow-up period. One patient (MJ, who also required one drop to control the IOP) ended up having a progressive myopic shift post surgery (-2.5 D) that stabilized after 3 months; nevertheless, the patient was satisfied with this outcome as it provided useful monovision. Table 1 summarizes each patient’s clinical details.

## DISCUSSION

The growth of the lens with the aging process plays an important role in mechanisms leading to PACG.^2^ Essentially, thickened lens contributes to primary angle closure. The benefit of phacoemulsification cataract surgery in controlling IOP in PACG cases has already been shown in previous studies.^2,11^ However, in extensive synechial angle closure this may not be sufficient to control IOP and 12% of such patients may need to have trabeculectomy in future.^12^

Goniosynechialysis is a technique that mechanically strips PAS from the trabecular meshwork to restore the trabecular function^4,13^ and improves the trabecular outflow facility.^4^ Normalization of IOP post-GSL depends on the function of trabecular meshwork. Prolonged PAS may lead to irreversible damage to the trabecular mesh-work, and it is therefore important to consider the likely duration that the PAS has been present before undertaking GSL.^13^ Concurrent removal of cataract with GSL improves vision and helps control IOP in PACG cases.^13^ The most common postoperative complications of GSL are transient, with hyphemia commonly being seen during stripping of PAS. The bleeding usually stops intraopera-tively and the hyphemia resolves spontaneously after the operation. More recently, endoscope assisted-GSL has been performed, which has many advantages including enhanced visualization, greater accuracy, and improved outcome compared with the conventional method as larger parts of the angle can be visualized and treated during endoscopic approach.^7,14^

Several studies have compared phacoemulsification with phacoemulsification plus ECP and found that the combined procedure can lower the IOP effectively and safely in glaucoma cases.^15-17^ The most common complication of ECP is fibrinous uveitis which resolves by intensive steroid therapy.^15^ Cystoid macular edema (CME) is seen more commonly in phaco-ECP compared with phaco alone.^18^ In our experience with phaco-ECP alone, both uveitis and CME occurred less frequently with the use of intracameral dexamethasone at the end of surgery. However, we did not encounter any such complications in our cases in the present study. One study showed that the refractive outcome is less predictable and there is a small myopic shift after phacoemulsification with ECP as opposed to those without ECP.^19^ Primary angle closure eyes have shorter axial lengths and anterior chamber depths; hence these eyes are prone to a myopic surprise postoperatively after phacoemulsification and IOL implantation.^20^ This is further exacerbated with ECP, possibly due to anterior rotation of ciliary processes.^19^ These same eyes are also susceptible to aqueous misdirection after phacoemulsification and IOL implantation leading to forward shift in the IOL position and a more myopic refractive outcome.^20^ A combination of these factors can play a role in any given case.

**Table 1:** **Table 1: **Patients’ clinical features

		*Diagnosis*		*PAS preoperative (degrees)*		*IOP*		*Patent peripheral iridotomy*		*Number of meds preoperative*		*IOP postoperative*		*PAS postoperative (degrees)*		*ECP*		*Number of meds*		*Follow-up postoperative (months)*	
Case 1 (MJ)		PACG		270		26		Present		3		18		20		*360*		1		12	
Case 2 (AG)		APAC		360		45		Present		2 + oral acetazolamide		10		20		360		0		12	
Case 3 (JN)		PACG		360		40		Present		4 + oral acetazolamide		10		0		360		0		12	

In studies that combined cataract extraction with ECP or with GSL, the need for IOP lowering medications was 19 and 94% respectively.^6,21^ Further glaucoma surgery was required in 4 to 14% of patients with extensive trabecular meshwork damage. In our small case series, two patients remained medication-free at the end of 1 year of follow-up and the third required just one hypotensive drop. This procedure was effective in one patient (JN) 2 years after the PI was done elsewhere and had ongoing angle closure, 360° synechial closure, and uncontrolled IOP on maximal medical treatment including acetazolamide.

In conclusion, we believe that combining phacoemul-sification with ECP and endoscopic GSL in patients with extensive synechial angle closure can maximize the IOP lowering effect by targeting inflow and outflow of aqueous humor concurrently. This should also reduce the need for future drainage surgery in these patients. To the best of our knowledge, this is the first report to use all of three procedures together; however, further studies with larger sample size, longer follow-up, and potentially a randomized control trial, are needed to understand the utility of this triple procedure in this potentially blinding condition.
